# Role of contractile prostaglandins and Rho-kinase in growth factor-induced airway smooth muscle contraction

**DOI:** 10.1186/1465-9921-6-85

**Published:** 2005-07-27

**Authors:** Dedmer Schaafsma, Reinoud Gosens, I Sophie T Bos, Herman Meurs, Johan Zaagsma, S Adriaan Nelemans

**Affiliations:** 1 Department of Molecular Pharmacology, University of Groningen, Antonius Deusinglaan 1, 9713 AV Groningen, The Netherlands

## Abstract

**Background:**

In addition to their proliferative and differentiating effects, several growth factors are capable of inducing a sustained airway smooth muscle (ASM) contraction. These contractile effects were previously found to be dependent on Rho-kinase and have also been associated with the production of eicosanoids. However, the precise mechanisms underlying growth factor-induced contraction are still unknown. In this study we investigated the role of contractile prostaglandins and Rho-kinase in growth factor-induced ASM contraction.

**Methods:**

Growth factor-induced contractions of guinea pig open-ring tracheal preparations were studied by isometric tension measurements. The contribution of Rho-kinase, mitogen-activated protein kinase (MAPK) and cyclooxygenase (COX) to these reponses was established, using the inhibitors Y-27632 (1 μM), U-0126 (3 μM) and indomethacin (3 μM), respectively. The Rho-kinase dependency of contractions induced by exogenously applied prostaglandin F_2α _(PGF_2α_) and prostaglandin E_2 _(PGE_2_) was also studied. In addition, the effects of the selective FP-receptor antagonist AL-8810 (10 μM) and the selective EP_1_-antagonist AH-6809 (10 μM) on growth factor-induced contractions were investigated, both in intact and epithelium-denuded preparations. Growth factor-induced PGF_2α_-and PGE_2_-release in the absence and presence of Y-27632, U-0126 and indomethacin, was assessed by an ELISA-assay.

**Results:**

Epidermal growth factor (EGF)-and platelet-derived growth factor (PDGF)-induced contractions of guinea pig tracheal smooth muscle preparations were dependent on Rho-kinase, MAPK and COX. Interestingly, growth factor-induced PGF_2α_-and PGE_2_-release from tracheal rings was significantly reduced by U-0126 and indomethacin, but not by Y-27632. Also, PGF_2α_-and PGE_2_-induced ASM contractions were largely dependent on Rho-kinase, in contrast to other contractile agonists like histamine. The FP-receptor antagonist AL-8810 (10 μM) significantly reduced (approximately 50 %) and the EP_1_-antagonist AH-6809 (10 μM) abrogated growth factor-induced contractions, similarly in intact and epithelium-denuded preparations.

**Conclusion:**

The results indicate that growth factors induce ASM contraction through contractile prostaglandins – not derived from the epithelium – which in turn rely on Rho-kinase for their contractile effects.

## Background

Growth factors have been reported to be involved in proliferation and differentiation of smooth muscle cells from a variety of tissues, including vasculature and airways [1,2]. In addition, several growth factors have been shown to induce contraction of vascular smooth muscle [3,4]. The mechanisms by which growth factors induce contraction have only been partly elucidated. Recent evidence has indicated that growth factor-receptors, such as the insulin-like growth factor-1 (IGF-1)-receptor, can activate the Rho/Rho-kinase pathway directly [5] and may be involved in smooth muscle contraction via Rho-kinase [6]. Smooth muscle contraction is mainly regulated by the phosphorylation level of the 20 kDa regulatory myosin light chain (MLC) [7]. MLC phosphorylation can be initiated by an increase in intracellular Ca^2+^-concentration ([Ca^2+^]_i_) followed by the Ca^2+^-calmodulin-dependent activation of myosin light chain kinase (MLCK). The extent of MLC phosphorylation is determined by the ratio of MLCK (MLC-phosphorylation) to myosin light chain phosphatase (MLCP)(MLC-dephosphorylation) activities [8]. Activated Rho-kinase mainly exerts its effect through inhibition of MLCP, resulting in an enhanced MLC phosphorylation and thus an increased level of contraction at a fixed [Ca^2+^]_i _(Ca^2+^-sensitization) [6,9].

In bovine airway smooth muscle, it has been demonstrated that prolonged incubation with growth factors modulates the phenotypic state of the muscle [10,11]. They have also been described to exert acute contractile effects on guinea pig tracheal smooth muscle [12,13]. Recently, we showed that growth factors are also capable of inducing human bronchial smooth muscle contraction. Thus, angiotensin II as well as IGF-1 induced a sustained contraction, which was completely dependent on Rho-kinase [14].

These observations may be of pathophysiological and pharmacotherapeutical interest, as expression levels both of growth factors (EGF)[15] and of receptors of growth factors (EGF[15], PDGF[15,16]) have been found elevated in asthmatic airways. Also, increased levels of PDGF have been found in exhaled breath condensate of asthmatic children with severe airflow limitation [17]. Moreover, previous studies showed an augmented role of Rho-kinase in acetylcholine induced bronchial smooth muscle contraction after repeated allergen challenge in rats [18,19]. Furthermore, we have recently demonstrated that the process of active allergic sensitization by itself, without subsequent allergen exposure, is sufficient to induce an enhanced role of Rho-kinase in guinea pig airway smooth muscle contraction *ex vivo *and airway resistance *in vivo *[20]. Therefore, a better understanding of the mechanisms by which growth factors induce a Rho-kinase dependent contraction is of pathophysiological and pharmacotherapeutical interest.

Epidermal growth factor (EGF) causes contraction of guinea pig tracheal smooth muscle via arachidonic acid metabolism in which presumably a tyrosine kinase and phospholipase A_2 _are involved [12,13]. It is well documented that receptor tyrosine kinases can activate mitogen-activated protein kinase (MAPK)/extracellular signal-regulated kinase (ERK)-kinase (MEK)[21-23]. Activation of MAPK by MEK may result in the activation of cytosolic phospholipase A_2 _(cPLA_2_) [24] and subsequent production of arachidonic acid and prostaglandins. Several studies have demonstrated that contractile prostaglandins are dependent on Rho-kinase [20,25]. Altogether, it can be hypothesized that growth factor-induced contraction is mediated via the MEK-dependent, cPLA_2_-mediated production of prostaglandins and subsequent activation of Rho-kinase. Therefore, we investigated the effects of inhibition of Rho-kinase, MEK and cyclooxygenase (COX) on growth factor-induced prostaglandin-production and contraction, using guinea pig tracheal smooth muscle preparations. In addition, we investigated the effects of selective prostaglandin receptor antagonists on growth factor-induced contraction.

## Methods

### Animals

Outbred specified pathogen-free male Dunkin Hartley guinea pigs (Harlan, Heathfield, U.K.), weighing 500–700 g, were used in this study. All protocols described in this study were approved by the University of Groningen Committee for Animal Experimentation.

### Isometric tension measurements

After experimental concussion and rapid exsanguination the trachea was removed and transferred to Krebs-Henseleit (KH) buffer solution (composition in mM: NaCl 117.5, KCl 5.6, MgSO_4 _1.18, CaCl_2 _2.5, NaH_2_PO_4 _1.28, NaHCO_3 _25.00 and D-glucose 5.55; pregassed with 95% O_2 _and 5% CO_2_; pH 7.4) at 37°C. The trachea was carefully prepared free of serosa and connective tissue. In some cases, the airway epithelium was carefully removed by moving a 15-cm woollen thread up and down the trachea twice. Epithelium denudation was confirmed by histological examination after fixating cryostat sections (5 μm) in acetone and staining with hematoxylin eosin. Single open-ring tracheal preparations were prepared and mounted for isometric recording, using Grass FT-03 transducers, in 20 ml water-jacketed organ baths (37°C) containing KH solution. During a 90 min equilibration period, with washouts every 30 min, resting tension was gradually adjusted to 0.5 g. Subsequently, the preparations were precontracted with 20 and 40 mM KCl. Following two wash-outs, maximal relaxation was established by the addition of 0.1 μM isoprenaline and tension was re-adjusted to 0.5 g, immediately followed by two changes of fresh KH-buffer. After another equilibration period of 30 min EGF (0.1, 1, 3, 10 or 30 ng/ml) or PDGF (0.1, 1, 3, 10 or 30 ng/ml) was applied or cumulative concentration response curves (CRCs) were constructed to stepwise increasing concentrations of histamine (1 nM – 100 μM), PGE_2 _(1 nM – 3μM) or PGF_2α _(1 nM – 10 μM). When maximal agonist-induced contraction was obtained, the tracheal rings were washed several times and maximal relaxation was established using isoprenaline. When used, the inhibitors of Rho-kinase (Y-27632, 1 μM), MAPK-ERK-kinase (MEK) (U-0126, 3 μM) or COX (indomethacin, 3 μM) were applied to the organ bath 30 min before agonist addition. This was also the case for the FP-receptor-and EP_1_-receptor-antagonists AL-8810 and AH-6809 (10 μM both, applied individually to separate preparations), respectively.

### Measurement of prostaglandin F_2α _and prostaglandin E_2 _production

Guinea pig tracheal rings were incubated using a 24-wells plate at 37°C. Each well contained 1 ml KH-buffer and 7 tracheal rings. Twenty-one rings were isolated from every trachea, so three conditions per preparation could be tested. Following a 30 min pre-incubation period, 100 μl of the medium was taken as the first sample. Subsequently, PDGF (10 ng/ml) was applied. To determine the time dependency of prostaglandin (PG)-production, samples were collected at 5, 10, 15, 20 and 30 min after PDGF-addition. Sampling was performed under a 95 % O_2 _/ 5 % CO_2 _atmosphere. PGF2α-and PGE_2_-production was determined using an ELISA-assay according to the manufacturer's protocol (R&D Systems, U.K.).

### Data analysis

All data represent means ± s.e. mean from *n *separate experiments. Statistical significance of differences was evaluated using either a one way analysis of variance (ANOVA) followed by a Bonferroni post-test or by a paired or unpaired two-tailed Student's t-test when appropriate, and significance was accepted when *P*<0.05.

### Chemicals

Platelet-derived growth factor AB (PDGF-AB, human recombinant) was from Bachem (Bubendorf, Switzerland) and epidermal growth factor (human recombinant), indomethacin, histamine dihydrochloride and (-)-isoprenaline hydrochloride were obtained from Sigma Chemical Co. (St. Louis, MO, U.S.A.). PGF_2α _was obtained from Pharmacia and Upjohn (Puurs, Belgium) and PGE_2 _was from BIOMOL (U.S.A). 1,4-diamino-2,3-dicyano-1,4-bis [2-aminophenylthio]butadiene (U-0126), (+)-(R)-trans-4-(1-aminoethyl)-N-(4-pyridyl) cyclohexane carboxamide (Y-27632) and 6-isopropoxy-9-xanthone-2-carboxylic acid (AH-6809) were obtained from Tocris Cookson Ltd. (Bristol, U.K.). 9α, 15R-dihydroxy-1 1β-fluoro-15-(2,3-dihydro-1H-inden-2-yl)-16, 17, 18, 19, 20-pentanor-prosta-5Z, 13E-dien-1-oic acid (AL-8810) was obtained from Cayman Chemical (U.S.A). All other chemicals were of analytical grade.

## Results

**Figure 1:**
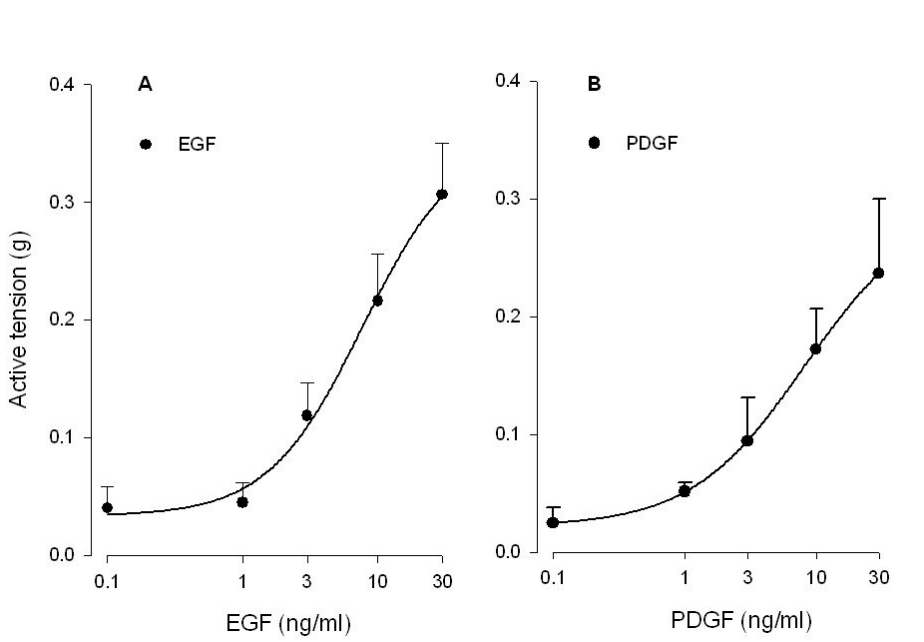
EGF (A)-and PDGF (B)-induced contraction of guinea pig open-ring tracheal smooth muscle preparations. Responses shown are corrected for basal myogenic tone, which amounted to 0.21 ± 0.06 g on average (0 % growth factor effect). Maximal effects were reached at concentrations of 30 ng/ml and amounted 0.31 ± 0.04 g (EGF, 100 % effect) and 0.24 ± 0.06 g (PDGF, 100 % effect), corresponding to 19.8 ± 2.8 % and 15.3 ± 4.1 %, respectively, of maximal histamine-induced contraction. Data represent means ± s.e. mean of seven (EGF) or four (PDGF) experiments, each performed in duplicate.

**Figure 2:**
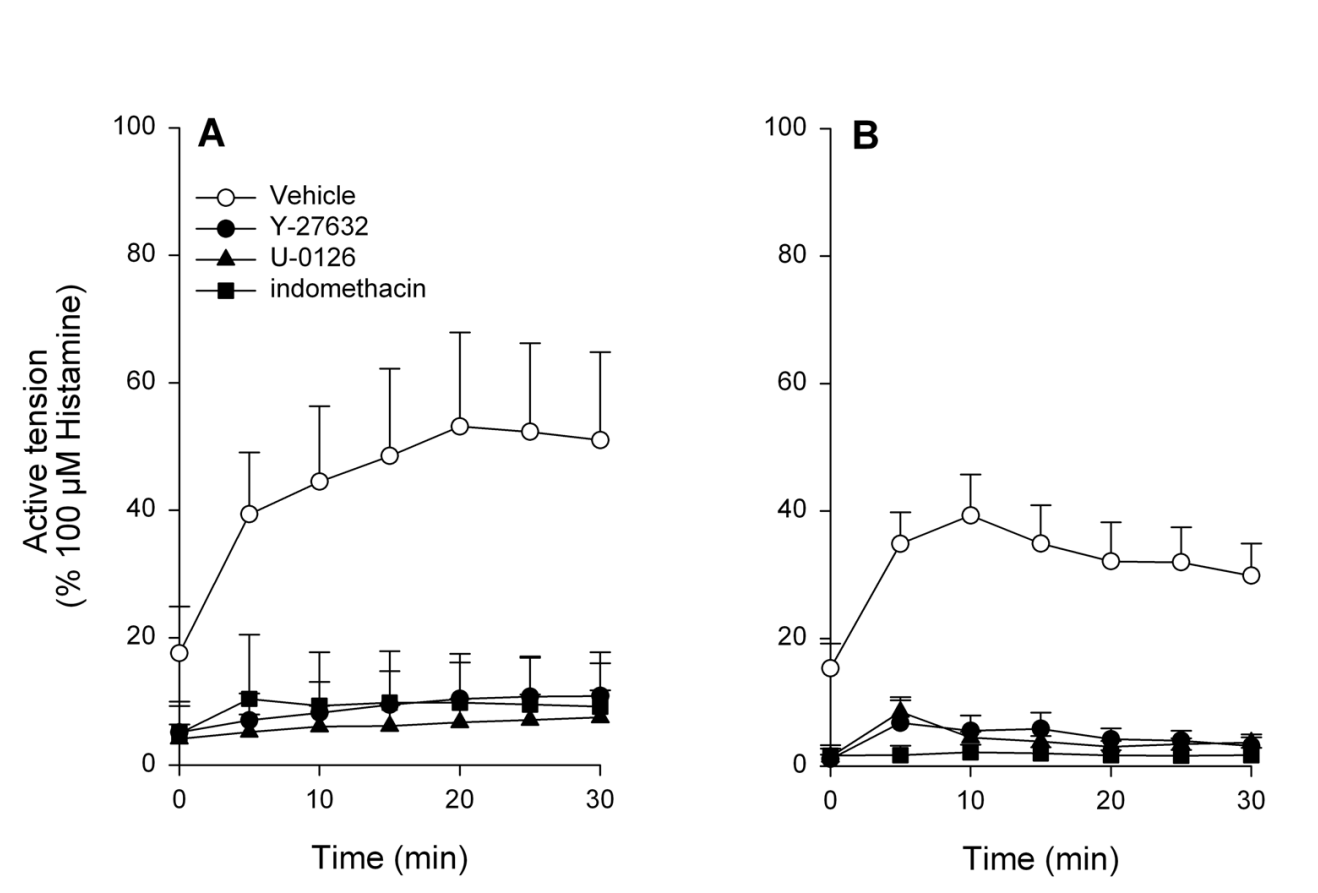
Effects of Y-27632 (1 μM), U-0126 (3 μM) and indomethacin (3 μM) on (A) EGF (10 ng/ml)-and (B) PDGF (10 ng/ml)-induced guinea pig trachealsmooth muscle contraction. Data represent means ± s.e. mean of five (EGF) or six (PDGF) experiments, each performed in duplicate.

To investigate the contractile effects of EGF and PDGF on guinea pig tracheal smooth muscle, CRCs of the growth factors were constructed (Fig. 1). Both EGF and PDGF were capable of inducing concentration-dependent contractions, with a potency (EC_50_) of 6.7 ± 2.3 ng/ml for EGF and 6.4 ± 2.8 ng/ml for PDGF. As shown in Fig. 2, both growth factors induced a slowly developing sustained contraction, which was prevented almost completely in the presence of either Y-27632 (1 μM), U-0126 (3 μM) or indomethacin (3 μM). Also, basal myogenic tone (expressed with respect to maximal relaxation established with isoprenaline) was abolished by these inhibitors (Fig. 2).

Since both MEK-(U-0126) and COX-inhibition (indomethacin) prevented growth factor-induced contraction, we envisaged that growth factor-induced prostaglandin production would be responsible for the observed contractions.

**Figure 3:**
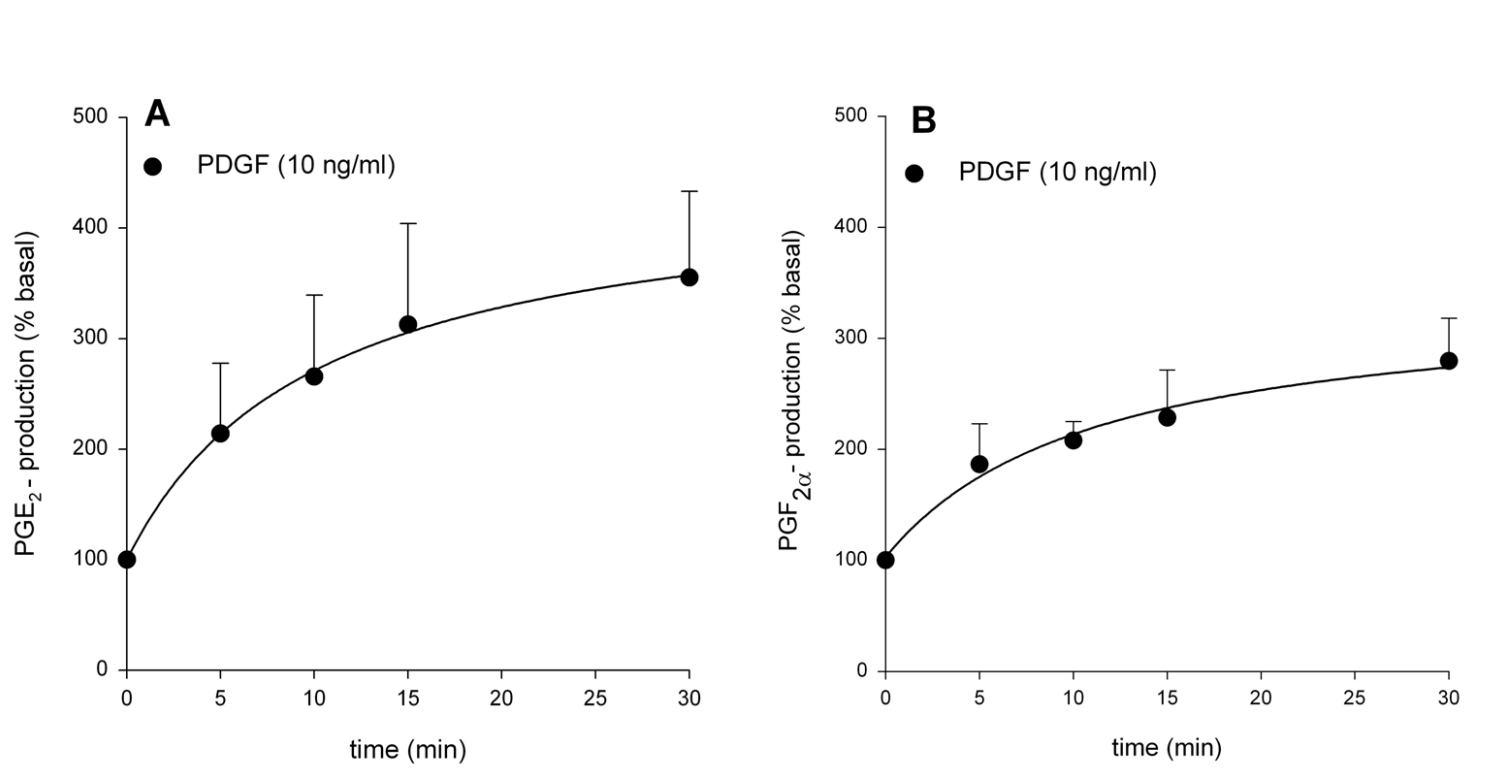
Growth factor-induced PGE_2 _(A) and PGF_2α _(B) release from guinea pig tracheal smooth muscle preparations. Basal release amounted to 963 ± 245 pg/ml (PGE_2_) and 1093 ± 204 pg/ml (PGF_2α_). Data represent means ± s.e.mean of five experiments.

**Figure 4:**
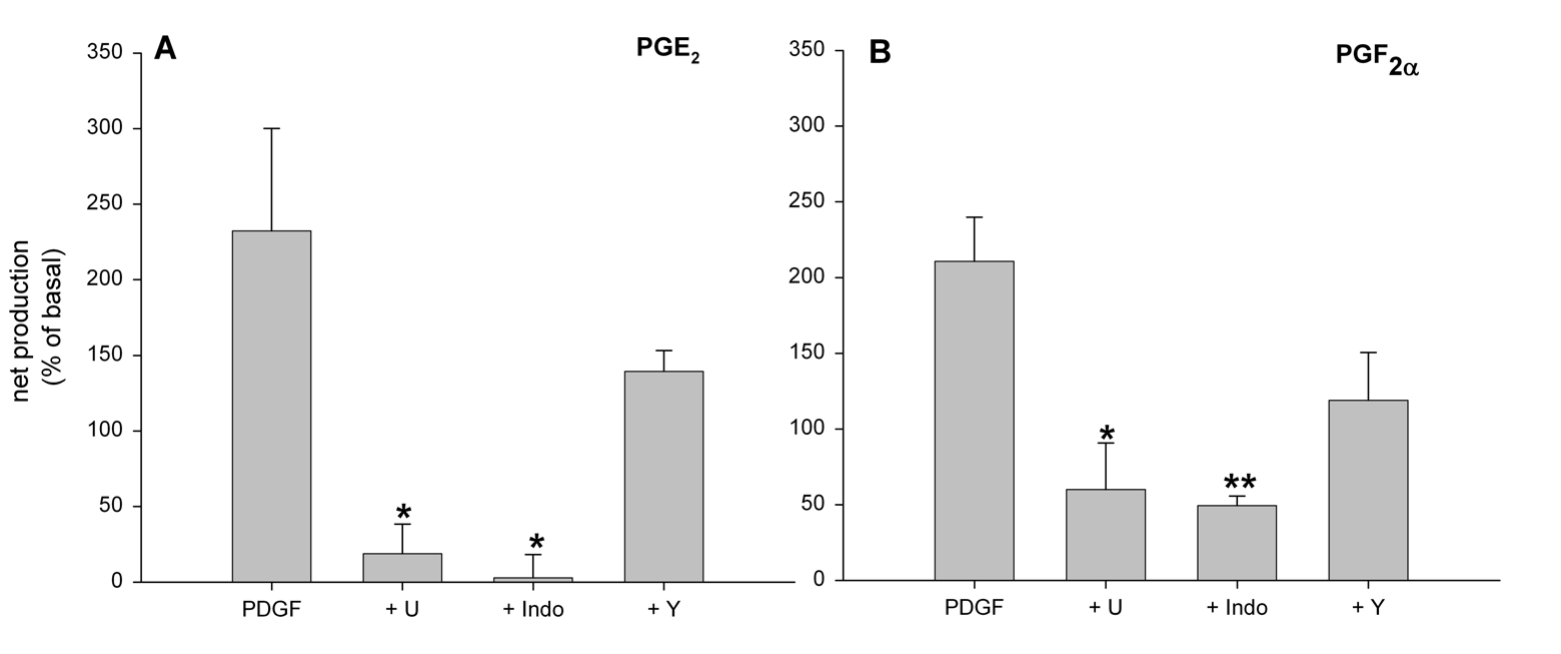
Effects of U-0126 (3 μM), indomethacin (3 μM) and Y-27632 (1 μM) on growth factor-induced PGE_2 _(A) and PGF_2α _(B) release. Data represent means ± s.e.mean of six (PGE_2_) and five (PGF_2α_) experiments. *p < 0.05, **p < 0.01 compared to PDGF.

**Figure 5:**
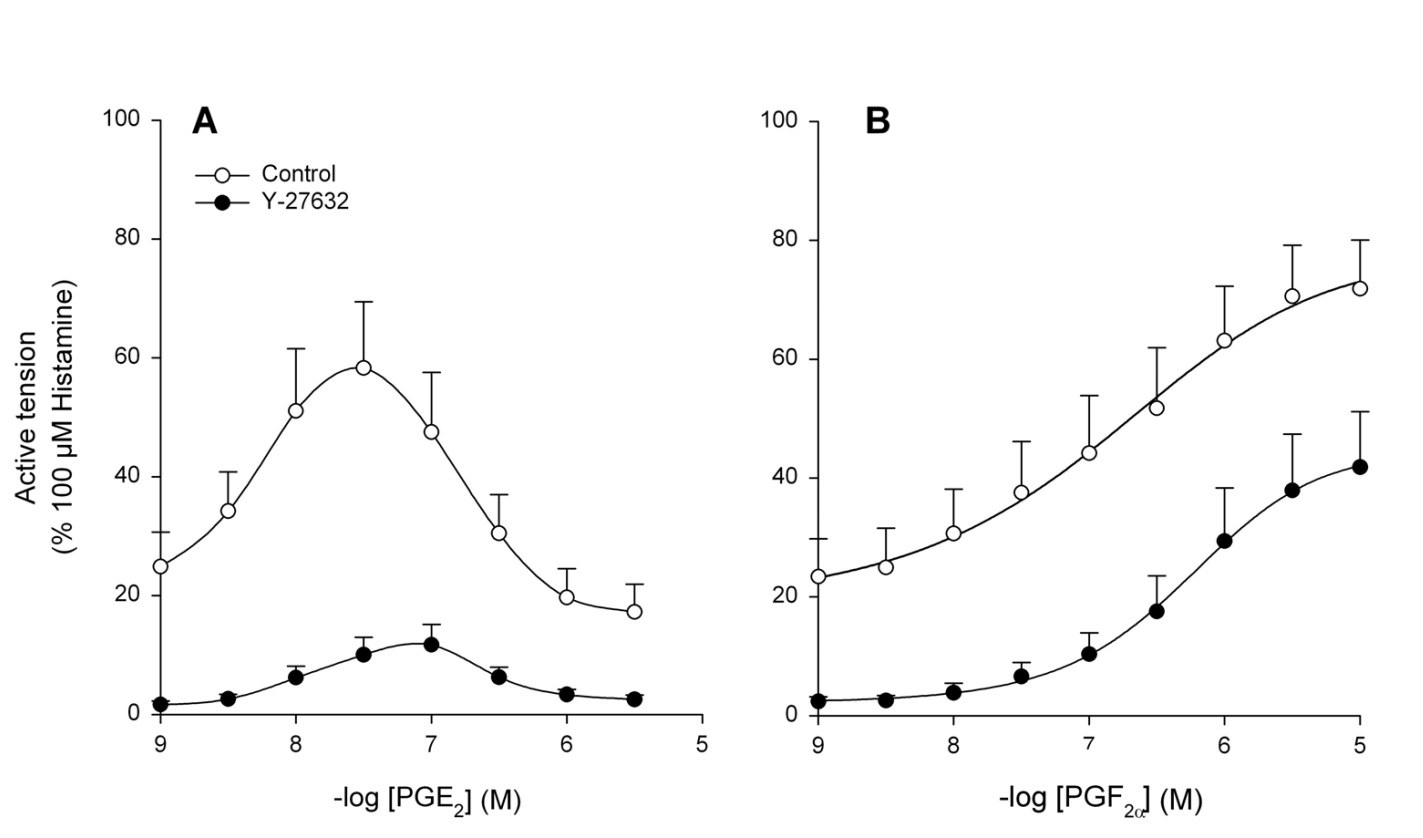
Effects of Rho-kinase inhibition on prostaglandin-induced contraction. PGE_2 _(A)-and PGF_2α _(B)-induced contraction in the absence and presence of Y-27632 (1 μM) of guinea pig open-ring tracheal smooth muscle preparations. Data represent means ± s.e.mean of four (PGE_2_) and seven (PGF_2α_) experiments, each performed in duplicate.

Stimulation of tracheal smooth muscle preparations with PDGF for 30 min greatly enhanced the release of prostaglandin E_2 _(PGE_2_) by 255 ± 78 % (from 963 ± 245 to 2762 ± 138 pg/ml; p < 0.01 at t = 30 min; Fig. 3A) and prostaglandin F_2α _(PGF_2α_) by 182 ± 38 % (from 1093 ± 204 to 2929 ± 570 pg/ml; p < 0.05 at t = 30 min; Fig. 3B). As shown in Fig. 4, both the release of PGE_2 _and PGF_2α _were significantly reduced in the presence of U-0126 (3 μM) and indomethacin (3 μM). In contrast to growth factor-induced contraction, no significant effect of treatment with Y-27632 (1 μM) was found on PGE_2 _(p = 0.23) or PGF_2α _(p = 0.08) release. These findings would suggest that prostaglandins produced in response to growth factor stimulation are capable of inducing a Rho-kinase-dependent contraction. Application of PGE_2 _caused ASM contraction in concentrations up to 0.03 μM (pEC_50 _= 8.22 ± 0.07, E_max _= 58.3 ± 11.2 %), but caused relaxation in higher concentrations (Fig. 5A). Indeed, Rho-kinase inhibition resulted in a decreased potency (pEC_50 _= 7.9 ± 0.2; p < 0.05) and maximal contraction (E_max _= 11.7 ± 3.5 %; p < 0.05) of PGE_2_-induced contraction. PGF_2α_-induced contractions (pEC_50 _= 6.8 ± 0.2; E_max _= 71.9 ± 8.2 %) were dependent on Rho-kinase as well, as indicated by the significantly decreased potency (pEC_50 _= 6.2 ± 0.2 ; p < 0.05) and maximal contraction (E_max _= 41.8 ± 9.3 %; p < 0.05) after treatment with Y-27632 (Fig. 5B).

**Figure 6:**
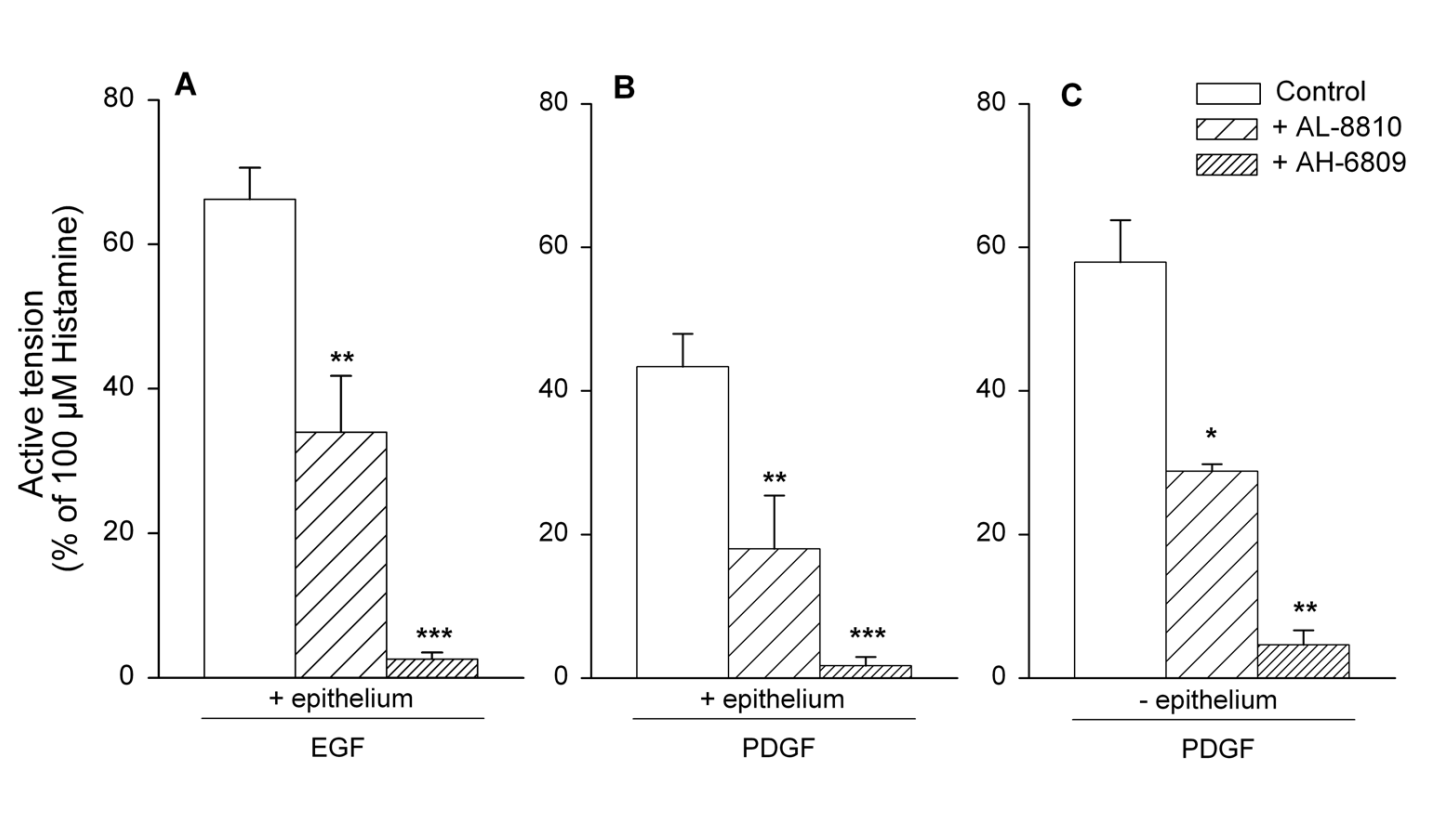
EGF (10 ng/ml, A)-and PDGF (10 ng/ml, B,C)-induced contraction of intact (A,B) and epithelium-denuded (C) guinea pig open-ring tracheal smooth muscle preparations in the absence or presence of AL-8810 (10 μM) or AH-6809 (10 μM). Data represent means ± s.e. mean of five (A,B) and three (C) experiments, each performed in duplicate. *p < 0.05, **p < 0.01 and ***p < 0.001 compared to control.

**Figure 7:**
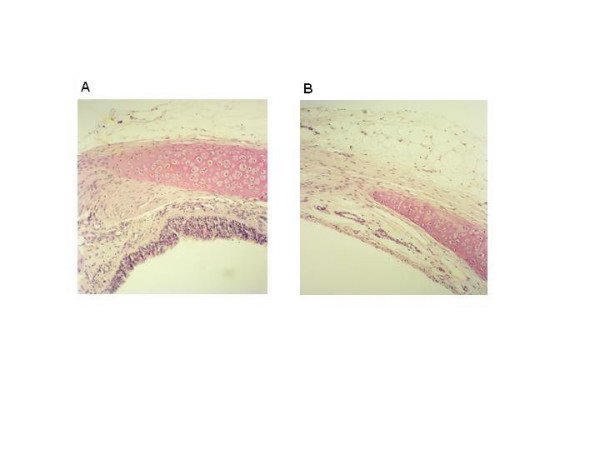
Representative photomicrograph of an intact (A) and epithelium-denuded (B) tracheal preparation. The photographs were taken at 100 × magnification.

To establish the functional contribution of the contractile PGE_2_-sensitive EP_1_-receptor and the PGF_2α_-sensitive FP-receptor to growth factor-induced contraction, the selective EP_1_-receptor antagonist AH-6809 (10 μM) and the selective FP-receptor antagonist AL-8810 (10 μM) were used. Both EGF-and PDGF-induced contractions were significantly reduced after treatment with AL-8810 (46,7 ± 13.0 % and 52.7 ± 13.2 % inhibition, respectively; p < 0.01 both), whereas contractions were almost abolished after treatment with AH-6809 (95.1 ± 3.1 % and 94.4 ± 4.7 % inhibition, respectively; p < 0.001 both)(Fig. 6A,B). To determine whether the epithelium was the source of the prostaglandins involved in growth factor-induced contraction, the effects of AL-8810 and AH-6809 on epithelium-denuded tracheal preparations were studied. Complete denudation was achieved as illustrated in Fig. 7. In these preparations, PDGF induced a slightly higher contraction compared to that in intact preparations, however the difference was not significant. Similar to intact preparations, PDGF-induced contraction was significantly reduced by both AL-8810 (48.8 ± 7.1 % inhibition; p < 0.05; Fig. 6C) and AH-6809 (92.1 ± 3.0 % inhibition; p < 0.01); Fig. 6C). Moreover, the inhibition in denuded preparations was very similar to that in intact preparations, both for AL-8810 and AH-6809, indicating that FP-and EP_1_-receptor stimulation involved in growth factor-induced contraction occurs independently of epithelium.

## Discussion

In this study we demonstrate that the growth factors EGF and PDGF induce contractions of guinea pig tracheal smooth muscle in a concentration dependent fashion. The concentration-effect range of EGF and PDGF (0.1 – 30 ng/ml) represents a pharmacological range very similar to other effects, such as mitogenesis of airway smooth muscle [26,10]. Since contractile effects of EGF have previously been associated with the production of eicosanoids [13] and contractions induced by of IGF-1 and angiotensin II appeared to be dependent on Rho-kinase [13,14], we analyzed whether contractions induced by submaximal concentrations of growth factors are dependent not only on Rho-kinase, but also on COX and MEK. This might be characteristic for growth factor-induced contraction, since potency and maximal contraction of histamine were shown to be independent of Rho-kinase, COX [20] and MEK (Schaafsma *et al*, unpublished observations) in guinea pig tracheal smooth muscle. Similarly, muscarinic receptor mediated contractions are only partially Rho-kinase-dependent [27,28], further illustrating the agonist-dependent role of Rho-kinase mediated calcium sensitization.

The role of Rho-kinase in growth factor-mediated effects could depend on the duration of growth factor stimulation. For instance, phenotypic modulation, as a consequence of 8 days stimulation with growth factors, or growth factor-induced proliferation of bovine tracheal smooth muscle, has been shown to be independent of Rho-kinase. However, in accordance with the effects of Rho-kinase inhibition on growth factor-induced contraction of human isolated bronchus [14], we demonstrate that Y-27632 fully inhibits growth factor-induced contraction of guinea pig tracheal smooth muscle. This indicates that growth factor-induced acute (smooth muscle contraction) and long term (e.g. modulation of smooth muscle phenotype) effects in airway smooth muscle may be differentially dependent on Rho-kinase.

Since MEK and COX inhibition almost abrogated growth factor-induced contraction, it can be suggested that growth factor-induced contraction relies on the production of prostaglandins. In several studies, it has been demonstrated that cytosolic phospholipase A_2 _(PLA_2_) can be activated in response to growth factors in a MAPK-dependent fashion, which results in subsequent arachidonic acid production [29-31]. In addition, contractile activity of EGF in guinea pig tracheal smooth muscle has been reported to be inhibited by indomethacin and by the phospholipase A_2 _inhibitor mepacrine [12]. As indicated by our results, PGF_2α _and PGE_2 _are being produced in response to PDGF-stimulation in a time-dependent fashion, similar to that of growth factor-induced contraction. Both prostaglandins are contractile agonists for airway smooth muscle [20,32,33]. Contractions induced by (exogenous) PGF_2α _and PGE_2 _were found to be largely dependent on Rho-kinase activity, which corresponds to observations in vascular smooth muscle [25,34], indicating that Rho-kinase plays an essential role in PGF_2α_-and PGE_2_-induced contractions. Interestingly, Rho-kinase inhibition had a more pronounced effect on PGE_2_-than on PGF_2α_-induced contractions. This can be explained, however, by realizing that the EP_2_-receptor mediated relaxation [35], as seen with the higher PGE_2_-concentrations, is suppressing the contractile phase more effectively when its Rho-kinase-dependent component is being inhibited.

In addition to direct contractile effects on guinea pig airway smooth muscle, PGF_2α _has been shown to augment cholinergic responsiveness of bovine airway smooth muscle [36], indicating an important role for PGF_2α _in regulating airway smooth muscle tone. PGF_2α _has been described to exert its contractile effects on smooth muscle through the FP-receptor [37,38]. Also, PGF_2α_-induced Ca^2+^-mobilization in vascular smooth muscle cells was dose-dependently inhibited by the selective FP-receptor antagonist AL-8810 [39]. In our study, a selective and effective concentration of AL-8810 [40,39] reduced EGF-and PDGF-induced contractions, indicating that PGF_2α _contributes to growth factor-induced contraction through the FP-receptor.

Smooth muscle contractions induced by PGE_2 _are predominantly mediated through activation of the EP_1_-receptor [41,32]. In guinea pig airway smooth muscle it has been previously found that PGE_2_-induced contractions could be dose-dependently inhibited by the EP_1_-receptor antagonist SC-19220 without modulating the relaxant activity (Van Amsterdam, 1991). Also, like PGF_2α_, PGE_2 _enhances cholinergic airway responsiveness of bovine airway smooth muscle [36]. In the present study we found that growth factor-induced contraction of guinea pig tracheal smooth muscle is essentially dependent on EP_1_-receptor stimulation, since the selective EP_1_-receptor antagonist AH-6809 [36] abrogated growth factor-induced contractions. Interestingly, these contractions were partially inhibited by FP-receptor blockade as well. From these observations, it may be hypothesized that PGF_2α_-mediated contractions partially rely on EP_1_-receptor stimulation (possibly by releasing small amounts of PGE_2_, selectively activating EP_1_-receptors) and that synergistic contractile effects of concomitant EP_1_-and FP-receptor stimulation occur.

Several growth factors, including EGF and PDGF, have been implicated in airway inflammation as they can be released from inflammatory cells, such as macrophages and eosinophils. Moreover, they can be derived from extravasated plasma, epithelial cells and the airway smooth muscle itself [2,42]. Growth factors are involved in tissue repair processes, therefore growth factor-induced contraction could protect damaged areas in the airways from the environment during these processes. In the pathophysiology of asthma, the repair process is usually not restricted to a single segment of the airways and growth factors may then contribute to airflow obstruction. Inhibition of such contractions might therefore be relevant under such pathophysiological conditions.

## Conclusion

**Figure 8:**
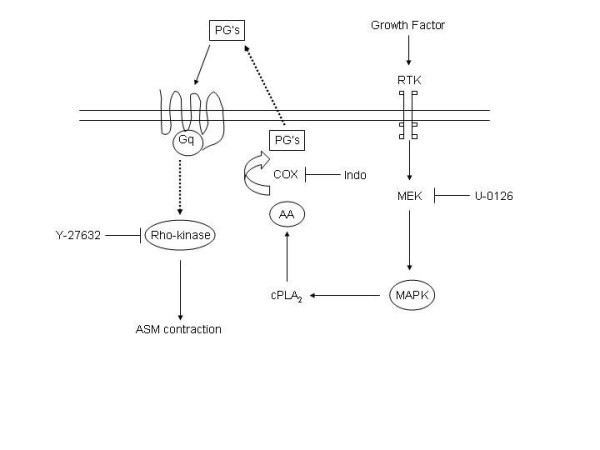
Putative mechanism of growth factor-induced airway smooth muscle contraction. Growth factors, like EGF and PDGF, bind to their receptors with intrinsic tyrosine kinase activity (RTK) and activate MAPK, which may result in increased levels of arachidonic acid (AA) via cytosolic phospholipase A_2 _(cPLA_2_) activation. As a consequence of cyclooxygenase (COX)-mediated conversion of AA, prostaglandins (PGs) are produced. These (contractile) prostaglandins, like PGF_2α _and PGE_2_, may in turn couple to their receptors and induce an airway smooth muscle contraction which is largely dependent on Rho-kinase. U-0126, indomethacin (indo) and Y-27632 are inhibitors of MAPK, COX and Rho-kinase, respectively.

Our overall results indicate that EGF and PDGF induce airway smooth muscle contraction through contractile prostaglandins. These prostaglandins are presumably produced by the consecutive actions of MEK, cytosolic PLA_2 _and COX and in turn are dependent on Rho-kinase for their contractile effects (Fig. 8). Since growth factor-induced contractions were inhibited by antagonists of contractile prostaglandin receptors both in intact and epithelium-denuded preparations, it can be concluded that the prostaglandins involved in growth factor-induced contraction are not primarily derived from the epithelium. Since both growth factors and increased Rho-kinase activity are associated with pathophysiological conditions and growth factor-induced contraction is fully Rho-kinase dependent, inhibition of Rho-kinase might be of therapeutical interest in the treatment of inflammatory (airway) diseases.

## Abbreviations

AA, arachidonic acid; AHR, airway hyperresponsiveness; ASM, airway smooth muscle; COX, cyclooxygenase; cPLA_2_, cytosolic phospholipase A_2_; CRC, cumulative concentration response curve; EGF, epidermal growth factor; EP_1_-receptor, prostaglandin E_2_-receptor type 1; FP-receptor, prostaglandin F_2α_-receptor; IGF-1, insulin-like growth factor-1; Indo, indomethacin; KH, Krebs-Henseleit; MAPK, mitogen-activated protein kinase; MEK, mitogen-activated protein kinase/extracellular signal-regulated kinase-kinase (MEK); pEC_50_, -log_10 _of the concentration causing 50 % of the effect; PDGF, platelet-derived growth factor; PG, prostaglandin; PGE_2_, prostaglandin E_2_; PGF_2α_, prostaglandin F_2α_; RTK, receptors with intrinsic tyrosine kinase activity

## Competing interests

The author(s) declare that they have no competing interests.

## Authors' contributions

DS designed and coordinated the study, performed a major part of the experiments, performed the statistical analysis and drafted the manuscript. RG participated in the design of the study, assisted in performing part of the experiments and contributed to the preparation of the manuscript. ISTB substantially assisted in performing the experiments. HM participated in the design of the study and the interpretation of the results. JZ participated in the design of the study, interpretation of results and final revision of the manuscript. SAN supervised the study, participated in its design and in the preparation of the manuscript. All authors read and approved the final manuscript.
